# Advancing Network Security with AI: SVM-Based Deep Learning for Intrusion Detection

**DOI:** 10.3390/s23218959

**Published:** 2023-11-03

**Authors:** Khadija M. Abuali, Liyth Nissirat, Aida Al-Samawi

**Affiliations:** Department of Computer Networks, College of Computer Sciences and Information Technology, King Faisal University, Al-Ahsa 31982, Saudi Arabia; 221445329@student.kfu.edu.sa (K.M.A.); lnissirat@kfu.edu.sa (L.N.)

**Keywords:** intrusion detection system, deep learning, CIC-IDS2018, support vector machines, multiclass classification

## Abstract

With the rapid growth of social media networks and internet accessibility, most businesses are becoming vulnerable to a wide range of threats and attacks. Thus, intrusion detection systems (IDSs) are considered one of the most essential components for securing organizational networks. They are the first line of defense against online threats and are responsible for quickly identifying potential network intrusions. Mainly, IDSs analyze the network traffic to detect any malicious activities in the network. Today, networks are expanding tremendously as the demand for network services is expanding. This expansion leads to diverse data types and complexities in the network, which may limit the applicability of the developed algorithms. Moreover, viruses and malicious attacks are changing in their quantity and quality. Therefore, recently, several security researchers have developed IDSs using several innovative techniques, including artificial intelligence methods. This work aims to propose a support vector machine (SVM)-based deep learning system that will classify the data extracted from servers to determine the intrusion incidents on social media. To implement deep learning-based IDSs for multiclass classification, the CSE-CIC-IDS 2018 dataset has been used for system evaluation. The CSE-CIC-IDS 2018 dataset was subjected to several preprocessing techniques to prepare it for the training phase. The proposed model has been implemented in 100,000 instances of a sample dataset. This study demonstrated that the accuracy, true-positive recall, precision, specificity, false-positive recall, and F-score of the proposed model were 100%, 100%, 100%, 100%, 0%, and 100%, respectively.

## 1. Introduction

The contemporary proliferation of technical breakthroughs has led to a heightened reliance on networks and applications in individuals’ everyday endeavors. Various sectors, such as business, education, healthcare, banking, and e-governments, exemplify the significant dependence on the Internet. Furthermore, the exponential expansion of social media platforms is engendering novel forms of hazards that extend beyond the virtual realm and manifest in tangible reality. Due to the increasing dependence on network infrastructure and the anticipated escalation in both the number and sophistication of attacks, traditional security techniques are considered insufficient in ensuring the necessary levels of security [1]. In contemporary society, online social networks have assumed a crucial role, being widely regarded as essential means of communication for individuals to connect with their loved ones. These platforms enable individuals to maintain contact and foster relationships, regardless of geographical barriers. Every day, substantial volumes of data, including photographs, videos, and concise messages, are sent via widely used social networking platforms such as Twitter, Facebook, Instagram, and YouTube, among others. The significance of social networks is growing due to the enormous volume of data they possess. Consequently, they are widely regarded as highly sought-after resources by hackers seeking to obtain individuals’ information without their explicit authorization. As a result, to counter attacks from hackers, who are also constantly evolving their attack detection techniques, there is a need for sophisticated and more effective techniques than before. The traditional systems used for identifying abnormal activity often use detection techniques based on event analysis and pre-set rules. They have drawbacks, including a lack of information on actual attacks, the cost of falsely detecting attacks in the field of security, and the inability to handle ever-updating attack techniques. Therefore, there is an urgent need for an automated system that learns from abnormal network traffic to detect new upcoming attacks. This paper aims to propose a support vector machine (SVM)-based deep learning system that will classify the data extracted from servers to determine the intrusion incidents in social media networks. The main reason that motivates us to conduct this study is that previous studies provided evidence that using deep learning in intrusion detection systems is deemed effective in detecting abnormal activity in large-scalable networks. Thus, this study proposed a deep learning-based intrusion detection system.

## 2. Related Works

At an early stage, intrusion detection systems were implemented using traditional techniques. Many studies have been conducted using statistical techniques to detect network intrusions. One of the earliest studies [2] built a statistical-based model using Six Sigma to estimate crucial network parameter thresholds. The suggested methodology provides a predetermined threshold to distinguish between normal, uncertain, and abnormal values for critical network variables and performs a comprehensive vulnerability assessment using these values. The statistical model had a prediction rate of 97%. In this study, the researchers conducted a comparative analysis of three statistical host-based intrusion detection system approaches: principal component analysis (PCA), Chi-square distribution, and Gaussian mixed distribution [3]. The outcome reveals that the PCA and Gaussian mixed distributions each have a detection rate of 97.5%, while the Chi-square distribution has a detection rate of 90%. The authors of [4] proposed a data mining-based intrusion detection system. The framework is based on the DTNB, which is a hybrid classifier (DTNB) that combines the naive Bayes (NB) and decision table (DT) methods. The accuracy result of the DTNB reached 97%. However, the main drawback of using intrusion detection-based statistical techniques is that they cannot handle large scalable networks.

The necessity to construct a proficient intrusion detection system employing contemporary technology arose as a result of the swift advancement of attackers’ methodologies in breaching computer networks. In the study conducted by the authors of [5], a model was developed to discern benign traffic from malicious communication using support vector machines (SVMs). The framework of the proposed model consists of numerous stages, including NSL-KDD dataset preprocessing, utilizing the information gain ratio to rank features (IGR), using a K-means classifier to create the optimal feature subset, and constructing an intrusion detection system based on support vector machines (SVMs). According to the study’s findings, the accuracy for 23 NSL-KDD features was 99.32%, while the accuracy for 30 features was 99.37%. In [6], the study employed an enhanced Bat algorithm using the K-means technique for feature extraction. Subsequently, the random forest algorithm was employed for categorizing, resulting in the generation of the ultimate output. The KDD Cup 1999 dataset was employed to assess the suggested model. Based on the findings of the study, the obtained accuracy was determined to be 96.42%, while the false-positive rate was observed to be 0.98%. Furthermore, the aforementioned study suggests the utilization of intrusion detection systems that employ random forest (RF) and support vector machine (SVM) algorithms [7]. The implementation of data preparation techniques in the NSL-KDD dataset was initially conducted. The study’s findings indicate that when all characteristics were utilized, SVM classifiers accurately categorized 90% of instances of DoS attacks, 89% of instances of Probe attacks, 79% of instances of R2L attacks, and 100% of instances of U2R attacks, after selecting the relevant features. In terms of accuracy, the RF classifier demonstrated varying performance across different attack types. Specifically, the accuracy rates for DoS, Probe, R2L, and U2R were 85%, 88%, 78%, and 100%, respectively, when considering all characteristics. The authors [8] developed a hybrid model that incorporates data mining approaches such as the K-means clustering method and the RBF kernel function, both of which are commonly employed in support vector machines (SVMs) for classification tasks. The KDD CUP 99 dataset was employed for training and evaluating the suggested model. The results of the study demonstrate that the suggested KMSVM model achieves an accuracy of 92.86% while utilizing all attributes. In comparison, KM achieves an accuracy of 86.67% and SVM achieves an accuracy of 40%.

Sistla, V. [9] built a network intrusion detection system using an SVM and a deep convolution neural network (DCNN) in which the model’s performance was evaluated using the NSL-KDD dataset. The experiment’s findings indicate that the accuracy was 96%. Similarly, Kim, J. and Shin, N. [10] used the KDD Cup 99 dataset to develop a deep neural network (DNN)-based intrusion detection system. The results demonstrated that the accuracy was 99%. Also, Nguyen, S. [11] proposed a deep learning-based intrusion detection system. The result demonstrates that the CNN model produced 99.87% detection accuracy. Similarly, Wang and Hong [12] presented a novel convolutional neural network-based method for detecting intrusions in the network. Where the accuracy of this model reached 97.7%. In addition, by using deep learning convolutional neural networks, Toupas and Chamou [13] and Liu, P. [14] produced accuracy higher than 99%. Similarly, in [15,16,17], the accuracy reached more than 99% when deep learning was used to detect intrusion in the network traffic. Furthermore, Maseer and Yusof [18] used support vector machines (SVMs) in the convolutional neural network (CNN) model. The proposed CNN model consists of an input layer that receives the data, convolution layers that create a features map from the data, and pooling layers that select the maximum values of the features map in the convolution layer; this is followed by another convolutional layer, another max pooling layer, a fully connected layer with a Relu activation function that learns and classifies outputs, and an output layer that represents the connection line as either normal or an attack. The models are tested on the CICIDS2017 dataset as part of the SVM classifier and are represented according to the factors involved in building the training models where the SVM model acts as a kernel function. The model produced an accuracy of 99.27%. In addition, Ref. [19] proposed an intrusion detection system (IDS) based on the convolutional neural network on CICIDS2017. The CNN model received an 8 × 8 matrix as the input. The topology consists of a convolution layer kernel size of 3 × 3, a max pooling layer with a kernel size of 2 × 2, a convolution layer kernel size of 3 × 3, and a max pooling layer with a kernel size of 2 × 2. Then, the pooled feature maps are converted into a 1D array in row order, which is the format expected by the fully connected layer. This generates an array of 288 elements for each input matrix. The study demonstrated that the accuracy of the proposed model reached 99.78%. In addition, the optimization of hyperparameters in one-dimensional (1D) convolutional neural networks (CNNs) for network intrusion detection with a genetic algorithm (GA) and particle swarm optimization (PSO) is examined in [20]. The model has been tested on the NSW-NB15 dataset. According to the study results, the PSO and GA produced accuracy of 99.28% and 99.31%, respectively. Also, in [21], the study provides a hybrid intrusion detection method that makes use of an optimized CNN by applying enhanced CNN parameters via the grey wolf optimizer (GWO) method, which improves the model’s prediction accuracy by fine-tuning the CNN parameters. By testing the model on the UNW-NB15 dataset, the accuracy reached 94.25%. Moreover, Ref. [22] developed an intrusion detection system using an artificial neural network and genetic algorithm. The experiments have been conducted on Bot-IoT, which consists of network traffic from different types of attacks. The results demonstrated that using a neural network with a genetic algorithm is effective in classifying network traffic and detecting abnormal traffic. 

Due to the rise of internet-based services, traditional tools have become inadequate for processing large volumes of network traffic. Therefore, there is an urgent need for an efficient and fast intrusion detection system that can process large amounts of complex network traffic. However, the previous work provides evidence that using deep learning in intrusion detection systems is deemed effective in detecting abnormal activity in large-scale networks. Using deep learning to analyze large-size network traffic can increase the detection accuracy and reduce false detection. In addition, support vector machines (SVMs) are the most used method for classifying network traffic. Thus, this research proposed a deep learning-based intrusion detection system using a support vector machine method for classifying network traffic on social media. This research is intended to fill the gap in research and investigate classification and intrusion detection in social media data.

The rest of this paper is organized as follows. Section 2 demonstrates related work on network intrusion detection using deep learning and machine learning methods. Section 3 describes the preprocessing procedure and data analysis. In Section 4, we describe the proposed architecture of the deep neural network. In Section 5, we present the results of our work, comparing it with related works. Finally, Section 6 gives a conclusion to this paper and presents future work.

## 3. Proposed Model

### 3.1. Data Preprocessing 

This section outlines the preprocessing techniques that were implemented on the dataset before training the proposed model. The CSE-CIC-IDS 2018 dataset undergoes several preprocessing techniques, including the removal of columns with zero values, dimensionality reduction, and down-sampling using principal component analysis (PCA), data normalization, and the enumeration of class tags. In some studies, such as [23], removing missing values, data normalization, and the enumeration of class tags are the only data preprocessing techniques that are applied to the dataset. However, the applied data preprocessing does not overfit the model during the training because after testing the model with new data, we see that the model is able to produce high detection accuracy. This provides evidence that the model is not overfitted because when the model is overfitted, it will not be able to produce high prediction results with the new data.

#### 3.1.1. CSE-CIC-IDS 2018 Dataset

**Figure 1:**
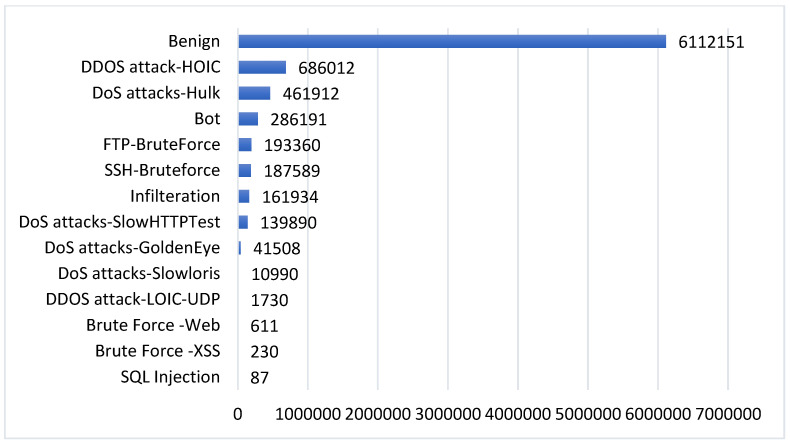
Dataset Classes Distribution.

This work relies on a public intrusion detection dataset, namely CSE-CIC-IDS 2018, which was created by The Canadian Institute for Cybersecurity (CIC) and the Communications Security Establishment (CSE) [24]. This dataset is synthesized to generate similar traffic to the real network traffic. It was generated by using the AWS computing platform to simulate the topology of a common LAN network. It contains 14 distinct attacks. These 14 classes and their distribution are shown in Figure 1.

The CSE-CIC-IDS2018 traffic is generated by the CICFlowMeter. The CICFlowMeter is a Java-written network traffic flow generator that provides greater flexibility in selecting and adding new features and better control over the length of the flow timeout. The statistical features, such as duration, number of packets, number of bytes, length of packets, etc., are also calculated separately in the forward and reverse direction because it generates bidirectional flows (Biflow), where the first packet determines the forward (source to destination) and backward (destination to source) directions. For feature extraction, the CICFlowMeter-V3 extracted 80 traffic features from the raw data and exported them as a CSV file. However, in this dataset, there is a set of columns that has zero values in all the entries. Also, the number of instances for each class is not balanced. In addition, the data are in different scales, with a high correlation between instances. For this, the study applied data preprocessing on the dataset before feeding it to the model.

#### 3.1.2. Data Preprocessing 

Deep learning algorithms have a direct correlation with the data they operate on, and to provide precise outcomes, it is imperative to preprocess the data. Data cleaning procedures are employed to eliminate correlation, eliminate redundancy, eliminate missing data, and balance classes. Data consistency is important in the field of deep learning for effectively processing and understanding data. Data that lack consistency are a significant challenge in training deep learning models [25].

**Missing data:** the CSE-CIC-IDS2018 dataset consists of 80 columns. Based on conducting a visual investigation, it was seen that a specific set of columns contains a value of zero for all entries. The columns with zero values have been removed. The columns that have been removed from the dataset are: bw_psh_flag, bw_urg_flag, fw_byt_blk_avg, fw_pkt_blk_avg, fw_blk_rate_avg, bw_byt_blk_avg, bw_pkt_blk_avg, and bw_blk_rate_avg. These columns have been removed from the dataset using the MATLAB function before feeding the data into the CNN model.

**Figure 2:**
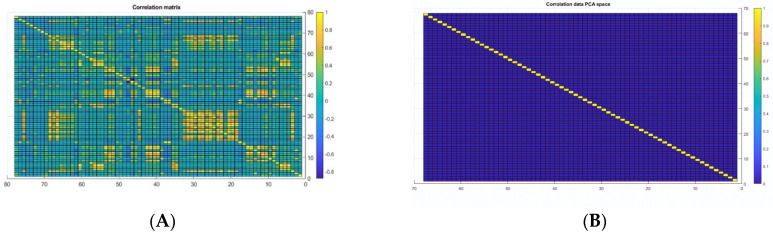
Correlation Matrix, (**A**) Raw Data 2D, (**B**) Row Data 3.

**Dimensionality reduction:** data normalization involves scaling down the data to be in the same range as the firing function, between 0 and 1 [26]. In the CSE-CIC-IDS2018 dataset, features are distinctive in their statistical properties, such as mean and standard deviation. Consequently, dimensionality reduction is implemented to bring all features into the same statistical range. The dimensionality reduction has been applied using the principal component analysis (PCA). In addition, PCA is also applied to eliminate the correlation and map the data into a low-dimensional space, where each feature is mapped into a PCA space, providing the most important information. Initially, we trained the model before applying dimensionality reduction, but the training accuracy could not exceed 70%. After a deep investigation of the dataset, we applied dimensionality reduction using principal component analysis. After applying PCA, the dataset size was reduced dramatically. The dataset had only 32 retained columns and 45 highly correlated features, which are considered redundant and have a minor contribution to the classification process. The original data in 2D, before applying PCA, are shown in Figure 2A, whereas the correlated data after applying PCA in 2D are shown in Figure 2B. PCA was applied to the dataset using the build-in layer in MATLAB, which defined the correlation of the data by calculating the eigenvector and eigenvalue of the covariance matrix. Then, the function was finding the major component by sorting these components by their eigenvalue in decreasing order. 

**Down-sampling:** in CSE-CIC-IDS2018, there are four classes with few instances, as shown in Figure 1. These four classes have less than 2000 instances, whereas the rest have more than 10,000 instances. To keep the balance between these classes, all classes with less than 10,000 instances have been removed. The four classes that were removed are SQL Injection, Brute Force-XSS, Brute Force-Web, and DDOS attack-LOIC-UDP. Therefore, this work considers ten classes, including nine attacks and benign traffic. Initially, the dataset had 17% attack traffic and 83% benign traffic. The down-sampling technique is applied to minimize the chance of overfitting and high bias on the network. For this, only 10,000 instances from each class are randomly chosen. Thus, the total number of instances for all classes will be 100,000, where the classes are completely balanced. These ten classes are: Benign, DDOS attack-HOIC, DoS attack-HULK, Bot, FTP-BruteForce, SSH-BruteForce, Infiltration, DoS attack-SlowHTTPTest, DoS attack-GoldenEye and DoS attack-Slowlorie. The down-sampling was implemented manually in the Excel file with the help of the filter feature. 

**Enumeration of class tags:** as we are implementing multilevel classification, the ‘Label’ column in the CSE-CIC-IDS2018 dataset was converted into numerical values for computational efficiency purposes. The class tags are shown in Table 1. Class tags were implemented with the help of the for-loop function in MATLAB, where each class in the for-loop is assigned to a specific class tag.

**Data splitting:** data splitting is frequently used in deep learning to separate data into train, test, and validation sets. The training dataset is a collection of instances that are used to fit the model’s parameters. The validation dataset is part of the dataset used to fit the model’s hyperparameters. The testing dataset is a part of the dataset used to evaluate the proposed model and detect the biasing on the network. The dataset splitting ratio used in this study is 50% for training, 20% for validation, and 30% for testing; this splitting ratio is one of the common splitting used in the literature [27]. The data splitting has been implemented in the MATLAB code where 50% of the dataset is taken as the training set, 20% is taken as the validation set and 30% is taken as the testing set. According to this splitting, each set of the dataset is used in different phases in training, validation and testing. 

### 3.2. Deep Neural Network Architecture

**Figure 3:**
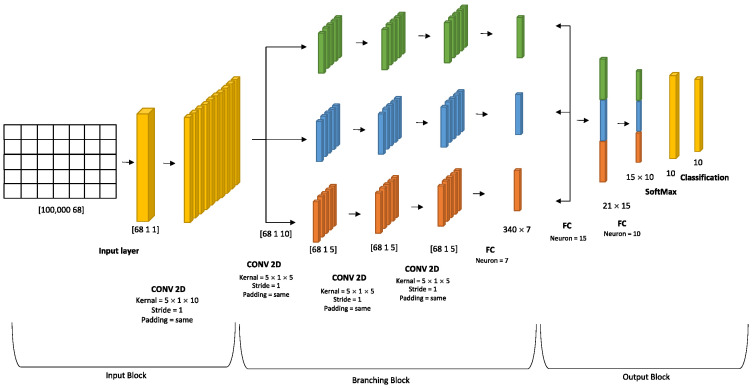
The Proposed Deep Learning System.

The proposed architecture for the deep learning system comprises an input layer, a series of hidden layers, and an output layer. The architectural structure is divided into three distinct blocks, namely the input block, which consists of the input layer and the general convolutional layer; the second block, which consists of three parallel branches that employ stacked convolutional layers followed by a fully connected layer to extract features; and the third block, which has two completely connected layers, namely the SoftMax layer and the classification layers, which are responsible for decision-making. In each block, a series of layers are present, whereby the convolution layers are responsible for feature extraction. The primary purpose of the pooling layer is to extract salient characteristics from the input data and effectively reduce its dimensionality. Nevertheless, the pooling layer may lead to the loss of important information within the data. For this, we employed a convolutional layer with a stride value of one to decrease the dimensionality of the feature instead of using the pooling layer [28]. In the process of feature extraction, the rectified linear unit (ReLU) activation function is utilized to activate every neuron inside the output of the network. The rectified linear unit (ReLU) is widely employed as an activation function to introduce nonlinearity into the system. The activation function layer serves as a crucial component within convolutional neural networks (CNNs), responsible for converting the input into a significant and interpretable representation of the data. However, to mitigate computational complexity and introduce parallelism to the data, the suggested architecture is partitioned into three parallel branches. Furthermore, the combination of several convolutional neural networks (CNNs) results in improved classification accuracy compared to individual CNNs [29]. This is because deep learning fusion has several benefits over traditional machine learning techniques. First, it can combine multiple data sources to create a more accurate and comprehensive understanding of data. This allows for more accurate predictions and better classification. Additionally, deep learning fusion can process large amounts of data quickly and efficiently, making it ideal for applications that require real-time analysis [30]. However, the three blocks of the proposed model will be discussed in detail in the following subsections. The proposed topology is shown in Figure 3.

A. Input Block: Within this section, a data input layer has been implemented to facilitate the insertion of network traffic into the convolutional neural network (CNN) model. Subsequently, a single two-dimensional convolutional layer was employed for feature extraction, consisting of ten kernels with dimensions of 5 × 1. The 2D convolutional layer is accompanied afterward by a batch normalization layer, which performs normalization on a mini-batch of data, independently across all observations for each channel. In this batch normalization layer, the topology is divided into three branches, and it is connected with the first convolution layer in the first, second, and third branches of the second block. The input block is shown in Figure 3.

B. Branching Block: The second block consists of three branches. These three branches are identical, including the same number of layers and kernels. Each branch starts from the batch normalization layer in the input block and ends with the depth concatenation layer in the output block. Each branch of the model utilizes three convolution layers, where each layer employs five kernels of size 5 × 1, and each convolution layer is followed by a batch normalization layer. At the end of each block, a fully connected layer with seven neurons is employed. The branching block is shown in Figure 3.

C. Output Block: This block has a depth concatenation layer, which takes inputs that have the same height and width and concatenates them along the channel dimension. This concatenation layer has three inputs to connect each fully connected layer at the end of each branch in the branching block with the output block. Subsequently, the architecture includes two fully connected layers. The first fully connected layer consists of 15 neurons, while the subsequent fully connected layer is composed of 10 neurons. These layers are utilized to classify 10 distinct classes. Following this, a SoftMax layer and a classification layer are employed to classify network traffic. The output block is depicted in Figure 3.

#### DAG SVM Algorithm

**Figure 4:**
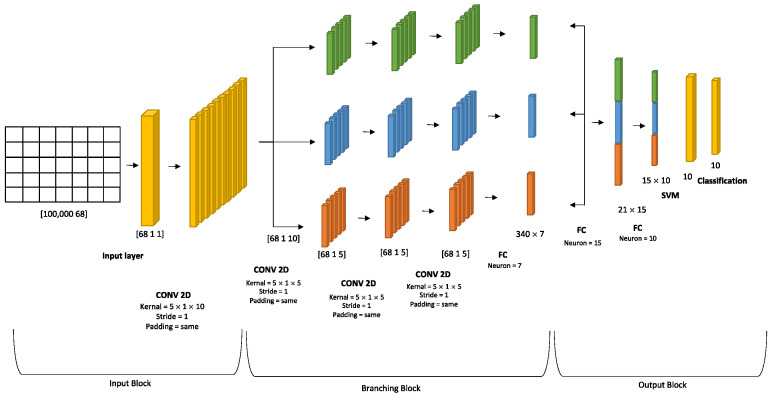
SVM Implementation.

This work aims to integrate an SVM with deep learning to develop IDS for social media. In SVM implementation, the SoftMax layer is replaced by the SVM layer. However, since the proposed architecture has been trained initially with the SoftMax layer, the SVM has been applied with the optimal activations (weight and bias) from the training. The reason for taking the activation instead of training the SVM again on the dataset is that the complexity will be higher in training the SVM again within the data. However, the kernel used for applying the SVM is a polynomial kernel [31]. The kernel function that has been applied in this work is:

f(x) = 〖(x − p)〗^q,(1)

where x is the data, and p and q are the constants that have been estimated during training. phase. The SVM topology is shown in Figure 4.

## 4. Experimental Setup

**Figure 5:**
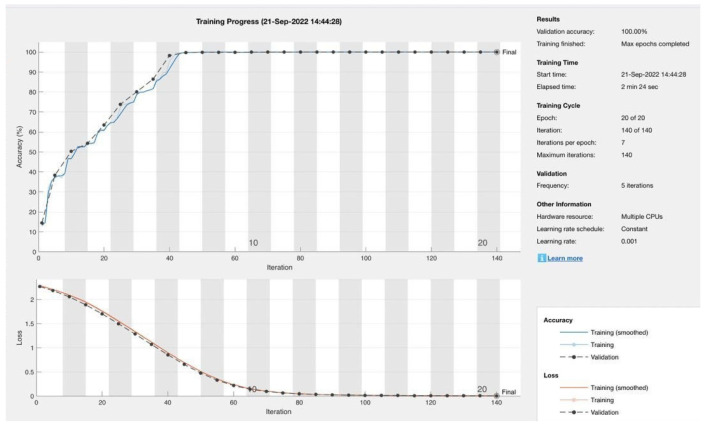
Training and Validation Process.

**Figure 6:**
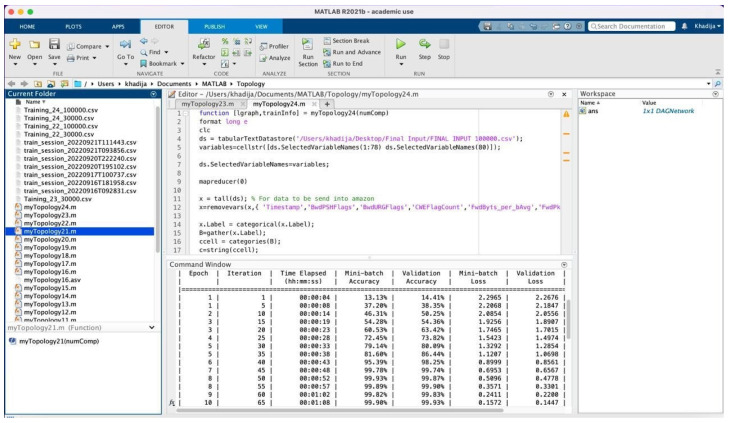
The Code with Training Details.

**Figure 7:**
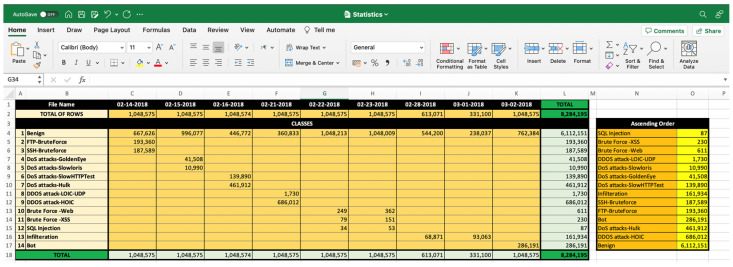
Preprocessing the Dataset.

Once the preprocessing approaches have been applied, the dataset is subsequently fed into the proposed model for training. Table 2 presents comprehensive information for the 15 training experiments that were carried out. The first round of experiments focused on 30 features extracted using PCA, which were treated as an independent feature called PCA space. This round consisted of three experiments. In the subsequent rounds, the number of features was expanded to 40, 50, 60, and finally 68. For each one of these feature numbers, experiments were conducted three times. Subsequently, the CNN model was enhanced by adding a support vector machine (SVM) layer, and subsequently, five experiments were conducted on 100,000 instances. Similarly, for model classification, there were five experiments conducted with different features ranging from 30 to 68 features, with 10 feature increments for each experiment. These experiments are carried out on a personal computer using the hardware and software characteristics listed in Table 3. The practical implementation was executed using MATLAB version R2022b, where the performance of the proposed deep learning model is assessed through the utilization of a confusion matrix. The training parameters utilized for optimizing weights and biases are associated with the Adam optimizer. The training parameters are presented in Table 4. However, developing and training the proposed model has some challenges. The first challenge is that training the model requires high computational resources. The second challenge is that applying the preprocessing technique and obtaining a high training accuracy takes a lot of trial and time. This challenge results in limitations, where the system is only tested on the CSE-CIC-IDS2018 dataset and has not been tested on another dataset. This limitation will be considered, and the system will be tested on another dataset in future work. The training and validation phase is shown in Figure 5, and part of the code with the training details is shown in Figure 6. Part of the preprocessing work is shown in Figure 7. 

## 5. Experimental Result

This section presents the result of the proposed architecture in terms of accuracy, true-positive recall, precision, specificity, false-positive recall, and F-score for each one of the 10 different classes that our model can detect. The evaluation of the model is based on a confusion matrix. The results will be discussed in detail in the following subsection.

A. Training and Validation Accuracy

**Figure 8:**
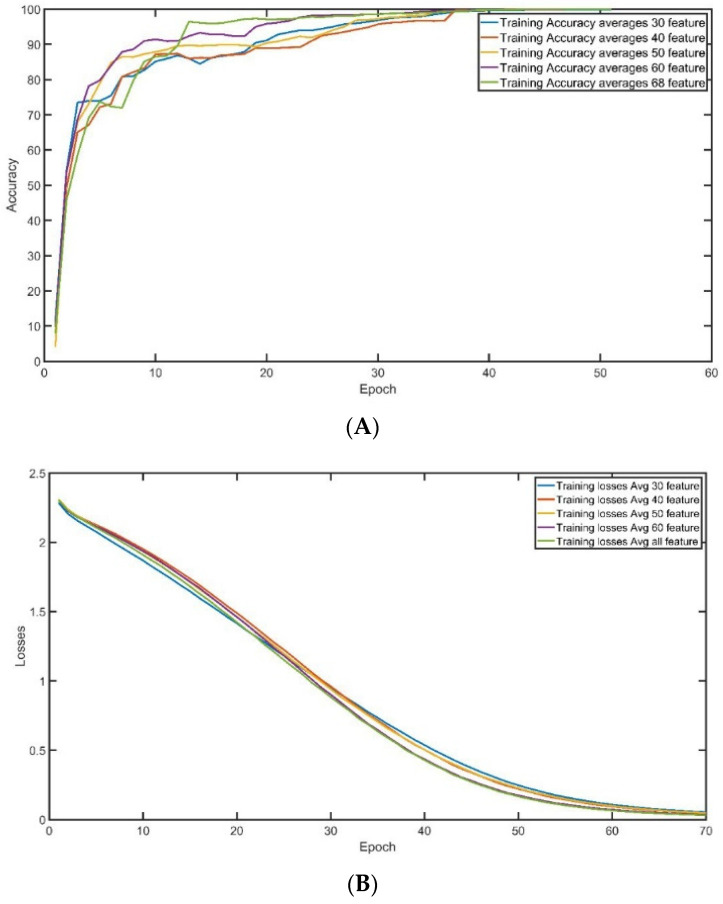
Training (**A**) Training Accuracy. (**B**) Training Loss.

The convergence of training accuracy is demonstrated in Figure 8A, which displays the average of three experiments conducted on the same dataset with 100,000 records and several features used for training. The accuracy of training surpasses 95% after 35 epochs, and the stability of training is generally excellent, regardless of the number of features employed. However, when all the features are chosen for training, there is a slight improvement in the convergence rate. Figure 8B exhibits the convergence of training loss during the training epoch, with each curve representing the average of three experiments run on the same data setting (100,000 records and a fixed number of features used for training). The training loss drops to less than 0.5% after 45 epochs, and the stability of training loss is generally good, regardless of the number of features used. However, there is a slight decrease in the loss convergence rate when 60 or all features are selected for training.

**Figure 9:**
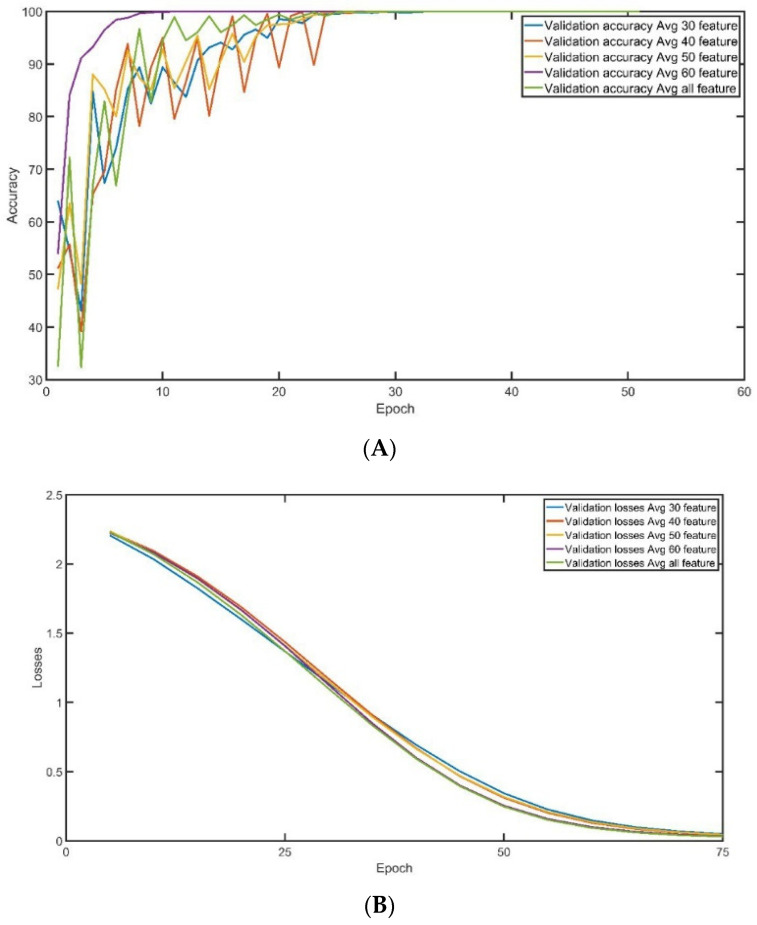
Validation: (**A**) Validation Accuracy. (**B**) Validation Loss.

Similarly, the convergence of validation accuracy during the training convergence is described in Figure 9A. Each curve represents the average of three experiments conducted using the identical data setting (100,000 records and the number of features used in training). After 30 epochs, the validation accuracy reaches more than 95%, regardless of the number of features employed. However, validation demonstrates a high oscillation at the beginning of the training until epoch 30. When 60 features are chosen for training, the rate of convergence is slightly improved. When 60 features are selected, the convergence is greater than 95% after 10 epochs. On the other hand, the convergence of validation loss during the training convergence is described in Figure 9B. Each curve represents the average of three experiments conducted using the identical data setting (100,000 records and the number of features used in training). After 45 epochs, the validation loss reaches less than 0.5%, and regardless of the number of features employed, validation loss demonstrates very high stability overall. When 60 or all features are chosen for training, the rate of validation loss is slightly reduced.

B. Classification Accuracy

**Figure 10:**
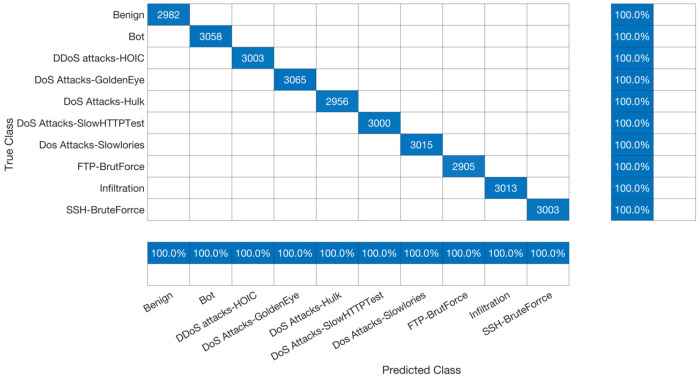
Confusion Matrix for 68 Features.

The accuracy of the proposed model when applied to 100,000 records using 30, 40, 50, 60, and 68 features is the same. Therefore, we have only presented the confusion matrix for the 68 features in Figure 10. Table 5 provides a detailed analysis of the accuracy of the confusion matrix for 100,000 records with 68 features. This study found that the proposed model performed well when applied to the CSE-CIC-IDS2018 dataset using 68 features and 100,000 records in all ten classes, including benign, bot, DDoS attack-HOIC, DoS Attack-GoldenEye, DoS Attack-Hulk, DoS Attack-SlowHTTPTest, DoS Attack-Slowlories, FTP-BruteForce, infiltration, and SSH-BruteForce. The accuracy, true-positive recall, precision, specificity, false-positive recall, and F-score for all classes were 100%, 100%, 100%, 100%, 0%, and 100%, respectively. Mainly, obtained 100% accuracy after applying the data preprocessing technique to the chosen synthesis dataset. The data preprocessing techniques had a major effect on our accuracy, where the accuracy before applying data preprocessing did not exceed 70% and the accuracy after applying data preprocessing reached 100%. This clarifies the reason that some studies produced varied results by applying the same model on the same synthesis dataset. This is because each researcher applies different data preprocessing techniques based on the researcher’s investigation of the chosen dataset. In other words, in this study, the accuracy did not exceed 70% before applying dimensionality reduction, whereas the accuracy reached 100% after removing the correlation and the duplication of the dataset using principal component analysis (PCA). In addition, the preprocessing techniques of the synthesis dataset depend on the researched investigation of the dataset. In other words, the preprocessing techniques applied to the synthesis dataset affect the accuracy result, and this preprocessing technique depends on the researcher’s investigation of the dataset itself. For example, in [32], we can see that the accuracy of using the same model on the same dataset after applying different preprocessing techniques is different. The reason is that the preprocessing techniques applied to the dataset affect the result even if the same model is applied to the same dataset. In addition, the main concept of overfitting is that the model is unable to produce good prediction results on the new data, whereas the proposed model was able to produce 100% accuracy on the new dataset. This provides evidence that the proposed model is not overfitted because it has high prediction capability on the new data. The consistent accuracy across different feature sets and the achievement of 100% accuracy after data preprocessing highlight the robustness of our model. While this performance pertains to the CSE-CIC-IDS2018 dataset, it is important to recognize that results may vary when applying the model to different datasets; this is a consideration we intend to address in our future work, further enhancing the model’s real-world applicability and generalizability. Additionally, the 0% false-positive recall signifies a low risk of false alarms, an essential factor for intrusion detection system reliability.

C. Support Vector Machine (SVM)

Although SoftMax achieved an accuracy of 100%, SoftMax was replaced with an SVM for the following reasons. Firstly, an SVM is more versatile than SoftMax in terms of its generalization capability. Secondly, an SVM provides better interpretability than deep learning methods like SoftMax. This is because an SVM takes into account the distribution of the data and probabilities, and also shows how the decision boundary divides the regions, making it easier to interpret the results. The SVM has been applied on 100,000 data sizes with 30 and 60 features.

**Figure 11:**
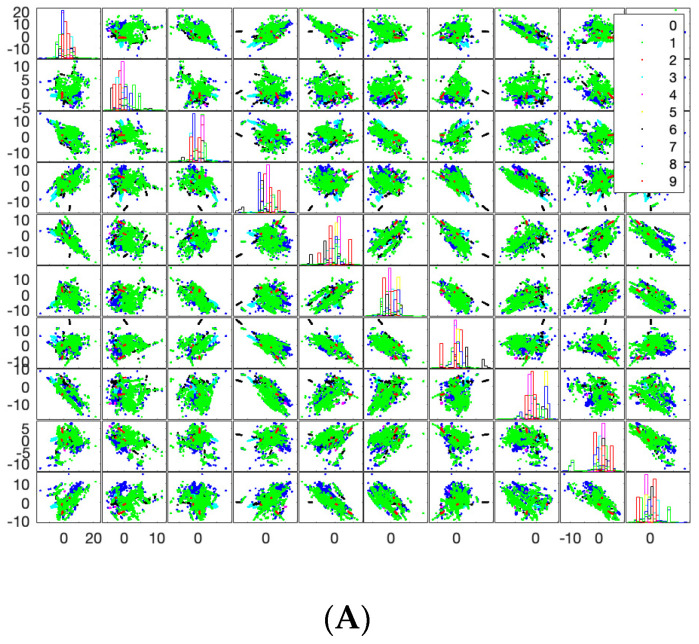

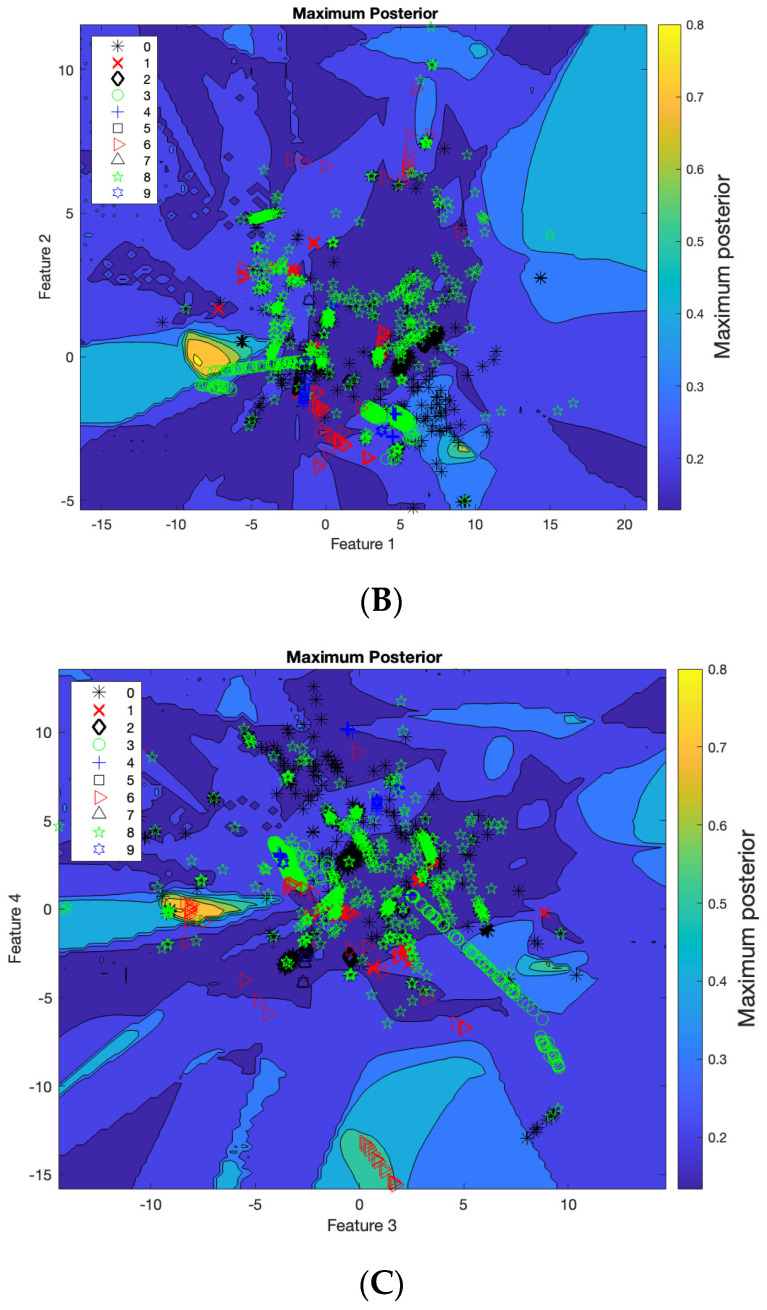
SVM on 100,000 Instances with 30 Features. (**A**) Scattered Matrix. (**B**) Feature 1 and 2. (**C**) Feature 3 and 4.

In terms of applying SVM on 100,000 rows with 30 features, Figure 11A shows a scattered matrix where the diagonal represents the probability distribution of each class in different projections on the dimensionality. The table includes 100 images that illustrate the scattered data and their projection in different dimensions. The columns and rows of the table represent the dimensionality of the data, ranging from 1 to 10. The 100 images show the scattered data and their projection for each combination of columns and rows. The probability distribution function of the data shows that there is at least one class that is well-defined and has its highest value in one of the dimensions. The scatter matrix that represents the data is symmetric, with the upper triangle being a mirror image of the lower triangle. As a result, all of the images in the table are mirror images of each other. The legend in the table provides the class tags that correspond to the data points in the scatter matrix.

Figure 11B shows the projection of the data on Feature 1 and Feature 2 in dimensionality. It shows the regions created by SVM projection in two dimensions. These regions are divided into contours to classify the classes. The contours in the figure distribute the probabilities of the maximum posterior, which range from 0.2 to 0.8, as shown on the right side. The colors in the figure represent the probability range and divide the regions into different probabilities to determine the various classes. Additionally, the figure illustrates that there is interference between the classes because it represents ten dimensions in a two-dimensional projection. However, combining the contour image in Figure 11B with the 100 scattered images in Figure 11A reveals that projecting data in different dimensions can help identify one or two classes. Figure 11A contains 100 images, each representing the projection of different features for each face of the 10 dimensions. Figure 11C demonstrates the projection of the data onto Feature 3 and Feature 4 in two dimensions.

**Figure 12:**
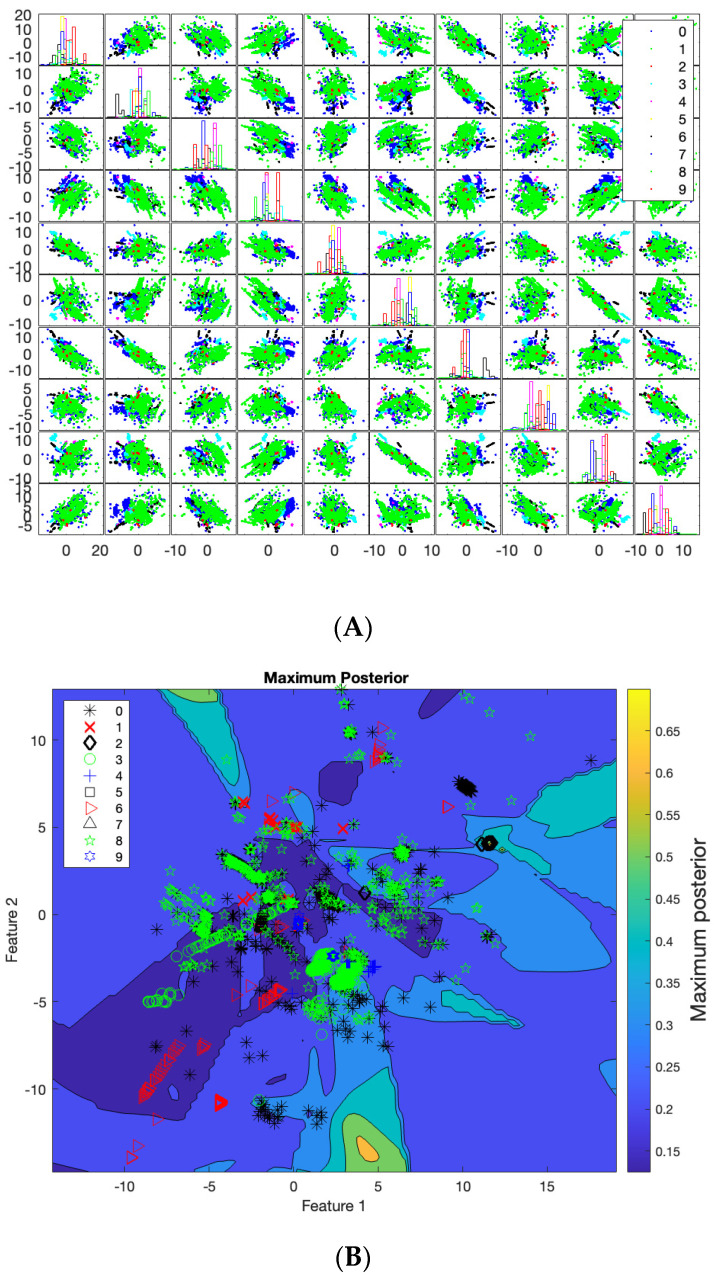

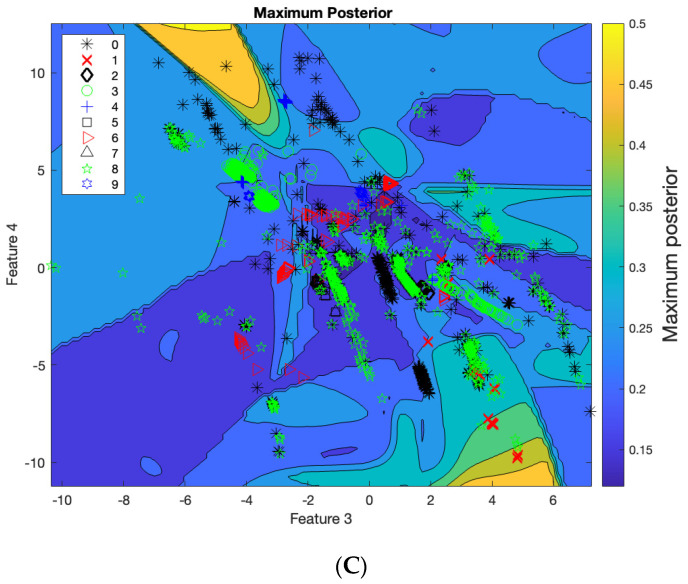
SVM on 100,00 Instances with 60 Features. (**A**) Scattered Matrix. (**B**) Feature 1 and 2. (**C**) Feature 3 and 4.

In terms of applying the SVM on 100,000 rows with 60 features, Figure 12A shows a scattered matrix where the diagonal represents the probability distribution of each class in different projections on the dimensionality. The table includes 100 images that illustrate the scattered data and their projection in different dimensions. The columns and rows of the table represent the dimensionality of the data, ranging from 1 to 10. The 100 images show the scattered data and their projection for each combination of column and row. The probability distribution function of the data shows that there is at least one class that is well-defined and has its highest value in one of the dimensions. The scatter matrix that represents the data is symmetric, with the upper triangle being a mirror image of the lower triangle. As a result, all of the images in the table are mirror images of each other. The legend in the table provides the class tags that correspond to the data points in the scatter matrix.

Figure 12B shows the projection of the data on Feature 1 and Feature 2 in dimensionality. It shows the regions created by SVM projection in two dimensions. These regions are divided into contours to classify the classes. The contours in the figure distribute the probabilities of the maximum posterior, which range from 0.2 to 0.8, as shown on the right side. The colors in the figure represent the probability range and divide the regions into different probabilities to determine the various classes. Additionally, the figure illustrates that there is interference between the classes because it represents ten dimensions in a two-dimensional projection. However, combining the contour image in Figure 12B with the 100 scattered images in Figure 12A reveals that projecting data in different dimensions can help identify one or two classes. Figure 12A contains 100 images, each representing the projection of different features for each face of the 10 dimensions. Figure 12C demonstrates the projection of the data onto Feature 3 and Feature 4 in two dimensions. The Table 6 is comparing the accuracy of the proposed model with the state of art approaches. 

## 6. Conclusions

A deep learning-based intrusion detection system using a support vector machine (SVM) was implemented for social media. The proposed model is capable of detecting abnormal behavior in the network and classifying the type of traffic between 10 different cases. During the data analysis and preprocessing of the dataset, the number of input features to the model was significantly reduced without compromising performance. The proposed model achieves a classification accuracy of 100% in multi-class classification problems. In future work, the model will be tested on another dataset to test the validity of the proposed model. Moreover, the model complexity will be further reduced by reducing the number of convolutional layers while maintaining good performance, which will also reduce training time for future experiments.

## Figures and Tables

**Table 1:** Class Tags.

Class Name	Class Tag
Benign	0
Bot	1
DDoS attacks-HOIC	2
DoS Attacks-GoldenEye	3
DoS attacks-Hulk	4
DoS attacks-SlowHTTPTest	5
DoS attacks-Slowlories	6
FTP-BruteForce	7
Infiltration	8
SSH-Bruteforce	9

**Table 2:** Number of Experiments.

	Number of Experiments				
Number of Features	Experiment 1	Experiment 2	Experiment 3	Experiment 4	Experiment 5
30	3				
40		3			
50			3		
60				3	
68					3
Total number of Experiments = 15					

**Table 3:** Hardware Specifications.

Items	Specification
Manufacturer City Country	MAC Foxconn Designed by California, assembled in China U.S
Model	MacBook Pro 2021
Processor Type	Apple M1 Pro chip
Installed Memory	Apple M1 Pro Chip, 16GB unified memory
Processor Speed	10-core CPU with 6 performance cores and 2 efficiency cores, 14-core GPU, 16-core Neural Engine, 200 GB/s memory bandwidth
Number of Cores	10-core CPU, 14-core GPU
Number of Threads	Single Thread
Machine Learning Tool	MATLAB (License Number: 40921045) version R2021b

**Table 4:** Experiment Parameters.

Parameters	Value
Algorithm	Adam solver
Max Epoach	50
Mini Batch Size	1000
ValidationFrequency	5
ValidartionPatient	10
ExecutionEnvironment	Parallel Pooling

**Table 5:** Confusion Accuracy Analysis for 100,000 Records of 68 Features.

Classes Labels	Accuracy	True-Positive Recall Sensitivity	Predicted Positive Precision	Actual Negative Specificity	False-Positive Recall Sensitivity	F-Score
**Benign**	100	100	100	100	0	100
**Bot**	100	100	100	100	0	100
**DDoS attacks-HOIC**	100	100	100	100	0	100
**DoS Attacks-GoldenEye**	100	100	100	100	0	100
**DoS Attacks-Hulk**	100	100	100	100	0	100
**DoS Attacks-SlowHTTPTest**	100	100	100	100	0	100
**DoS Attacks-Slowlories**	100	100	100	100	0	100
**FTP-BrutForce**	100	100	100	100	0	100
**Infiltration**	100	100	100	100	0	100
**SSH-BruteForrce**	100	100	100	100	0	100

**Table 6:** Comparing the Proposed System with State-of-the-Art Approaches.

References	Method	Dataset	Accuracy
[23]	Deep Convolution Neural Network (DCNN)	CSE-CIC-IDS2018	95%
[33]	Deep Convolution Neural Network (DCNN)	CSE-CIC-IDS2018	99.98%
[34]	Apache Spark, CNN	CSE-CIC-IDS2018	Apache Spark produced 100% for all classes. The CNN produced 98% for two classes and 100% for the other classes.
**Proposed System**	Deep Convolution Neural Network (DCNN)	CSE-CIC-IDS2018	100%

## Data Availability

No new data were created or analyzed in this study. Data sharing is not applicable to this article.
